# Job Selection After Orthopedic Surgery Training: Why Are Our Trainees Failing to Select the Right Job?

**DOI:** 10.7759/cureus.5539

**Published:** 2019-08-31

**Authors:** Joseph L Laratta, Jeffrey L Gum, Jamal N Shillingford, Hemant Reddy, Ronald A Lehman, Charles H Crawford, Steven D Glassman, Leah Carreon

**Affiliations:** 1 Orthopaedics, Norton Healthcare, Norton Leatherman Spine Center, Louisville, USA; 2 Orthopaedics, Northeast Ohio Medical University (NEOMED), Rootstown, USA; 3 Orthopaedics, Columbia Spine Hospital, New York, USA; 4 Orthopaedic Surgery, University of Louisville, Louisville, USA; 5 Research, Norton Healthcare, Norton Leatherman Spine Center, Louisville, USA

**Keywords:** systems-based practice, orthopedic surgery training, medical education, job selection, job attrition

## Abstract

A survey was administered to a random sampling of the American Academy of Orthopaedic Surgeons (AAOS) members to determine the rate at which recently trained orthopedic surgeons switch their first job and to identify factors affecting the job selection process.

There were 351 (21%) respondents. Respondents considered practice location (41%), practice type (28%), and family proximity (23%) as most important while research opportunity (54%) and signing bonus (33%) were considered least important in their first job. Half of the respondents (51%) left their first job before the completion of their fifth year; most left for financial reasons (34%) or because the practice was not as advertised (31%). Many (53%) stated they had minimal training in selecting their first job and most (88%) felt inadequately prepared for the business side of orthopedics. Further studies are needed to evaluate the high rate of initial post-training job attrition to decrease the personal and societal costs of this phenomenon.

## Introduction

Professional and financial stresses accompany the training of an orthopedic surgeon. Anecdotally, more than half of orthopedic trainees switch jobs within a few years of starting practice. Furthermore, orthopedic surgeon burnout ranges from 32% to 59.6% [1-2] Despite this, orthopedic surgeons have greater job satisfaction as compared to general surgeons [2]. It remains unclear as to why a large proportion of orthopedic surgeons change initial jobs.

The primary purpose of this study was to determine what orthopedic surgeons consider the most important factors in the job selection process and what factors are associated with changing jobs so quickly after completing training. This may help future orthopedic trainees navigate a successful career pathway by addressing the job attrition phenomenon, allowing them to make well-informed decisions [3-4].

## Materials and methods

A web-based survey was developed using SurveyMonkey, a platform for online data collection (California, US). The survey consisted of 26 questions, collecting data on demographics, details regarding the respondent’s first and second jobs, and specifics of the job-switch process. A link to the survey was emailed to all 1,656 US-based members of the American Academy of Orthopaedic Surgeons (AAOS) between August and September 2017 with 311 (19%) physicians responding.

Statistical analyses were performed using SPSS (IBM v24.0, Armonk, NY, US). Independent sample t-tests were used for continuous data. Fisher exact tests were used for categorical variables. Associations between variables were examined using Pearson correlations. Statistical significance was defined as p<0.05.

## Results

Of the 311 respondents, half were still at their first job and the other half had already switched jobs. Around half (46%) were more than 15 years post-training, 20% were 10-15 years, 22% were five to 10 years, and 12% were less than five years post-training (Table 1).

**Table 1:** Training and practice type of respondents

Training and practice type of respondents				
	Yes	No	Total	p-value
N	154 (50%)	157 (50%)	311	
Years post-training				0.016
0-1 years	2 (1%)	0 (0%)	2 (1%)	
1-2 years	5 (3%)	2 (1%)	7 (2%)	
2-3 years	3 (2%)	1 (1%)	4 (1%)	
3-4 years	9 (6%)	3 (2%)	12 (4%)	
4-5 years	10 (6%)	6 (4%)	16 (5%)	
5-10 years	40 (26%)	27 (17%)	67 (22%)	
10-15 years	29 (19%)	32 (20%)	61 (20%)	
>15 years	56 (36%)	86 (55%)	142 (46%)	
Fellowship-trained	136 (88%)	131 (83%)	267 (86%)	0.256
Practice type				0.802
Private (same specialty group)	74 (48%)	70 (45%)	144 (46%)	
Academic	27 (18%)	31 (20%)	58 (19%)	
Hospital-employed	18 (12%)	13 (8%)	31 (10%)	
Private (multi-specialty group)	15 (10%)	14 (9%)	29 (9%)	
Other (please specify)	9 (6%)	12 (8%)	21 (7%)	
Private (solo)	7 (5%)	12 (8%)	19 (6%)	
Hybrid	4 (3%)	5 (3%)	9 (3%)	

As expected, a larger proportion of respondents that have been in practice longer had switched jobs (p=0.016). The majority of surgeons completed fellowship training (85%), which was not associated with switching jobs (p=0.256). Almost all the respondents who did not complete fellowship were 15 years (38) or 10-15 years (6) post-training. Single-specialty private group practice accounted for 46% of first jobs, followed by academic (19%), hospital-based (10%), and private practice in a multispecialty group (9%).

Respondents were asked to rank the factors (1=most important to 7=least important) they considered while deciding on both their first job and second jobs. The factors that respondents considered to be important for their first job were similar between those who changed and those that did not change their first job (Table 2).

**Table 2:** Factors ranked by importance (1=most important to 7=least important) in selecting first and second jobs

Factors ranked by importance (1=most important to 7=least important) in selecting first and second jobs			
	First Job		Second Job
	Yes	No	
N	154	157	157
Ranked by importance			
Geography (location)	2.2	2.2	2.5
Practice type	2.5	2.8	2.4
Family proximity	3.0	3.4	3.5
Potential income	3.6	3.8	3.7
Starting income	4.5	4.1	4.0
Signing bonus	6.1	5.9	6.0
Research opportunity	6.1	5.8	5.9

The most important considerations were location, practice type, and family proximity. For those that switched jobs, practice type became a more important consideration than location. Most respondents did not stay close to their residency or fellowship program either for their first or second jobs (Table 3).

**Table 3:** Distance from training program to first and second jobs

Distance from training program to first and second jobs					
	Proximity to first job				
Proximity to second job	Joined training program	0-50 miles	50-150 miles	>150 miles	
Joined training program	1	1	0	5	
1-50 miles	1	8	1	5	
50-150 miles	0	1	6	4	
150 miles or more	3	6	2	77	

Only a small proportion of respondents answered that they had adequate guidance in terms of job selection during their training, and this proportion was similar between those that switched (12%) and those that did not (12%) switch jobs (Table 4).

**Table 4:** Amount of business education respondents received during training

Amount of business education respondents received during training				
	Still at First Job			
	Yes	No	Total	
Guidance during training				
None	36 (23%)	67 (43%)	103 (33%)	0.001
Minimal	92 (60%)	71 (45%)	163 (52%)	
Adequate	26 (17%)	19 (12%)	45 (14%)	
Number of contracts reviewed				
0	7 (5%)	16 (10%)	23 (7%)	0.235
1-2	76 (49%)	75 (48%)	151 (49%)	
3-4	64 (42%)	55 (35%)	119 (38%)	
5-6	5 (3%)	6 (4%)	11 (4%)	
>6	2 (1%)	5 (3%)	7 (2%)	
Contract review				
Contract attorney	88 (57%)	87 (55%)	175 (56%)	0.168
No one	31 (20%)	44 (28%)	75 (24%)	
Other	35 (23%)	26 (17%)	61 (20%)	

The proportion of respondents who answered that they had no guidance was smaller in those who were still at their first job. The proportion of respondents who sought the assistance of a contract lawyer was similar between the two groups. Interestingly, the respondents who had answered that they had no guidance also had a smaller number who consulted a contract attorney and a larger number who did not consult anyone before signing their first contract (Table 5).

**Table 5:** Amount of business education and contract review

Amount of business education and contract review				
	Amount of business education			
Contract reviewed with	None	Minimal	Adequate	0.000
Contract attorney	43 (42%)	101 (62%)	31 (69%)	
No one	40 (39%)	28 (17%)	7 (16%)	
Other	20 (19%)	34 (21%)	7 (16%)	

## Discussion

This is the first study to identify factors that lead to a recently trained orthopedic surgeon choosing their first job as well as factors that lead to switching jobs. About half of the respondents switched jobs. As expected, respondents who more recently finished their training were still at their first jobs, as most respondents switched jobs within five years after their training.

Whether or not they switched jobs, practice location and practice type were the most important factors in choosing their first job post-training. For those that switched jobs, these two were still the most important factors, but practice type was more important than practice location. The finding that one of the more common reasons for leaving their first job was that the practice was not as advertised is consistent with this finding, possibly indicating that surgeons learned from the reasons they left their first job or found more suitable second jobs. However, the proportion of respondents within each practice type were similar between those who switched and those who did not switch jobs. This may be reflective of the availability of these practice types and not necessarily the choice of the respondents.

It is clear that the majority of trainees did not see any value in staying close to their residency or fellowship program when considering their job opportunities and that the choice of practice location probably includes other factors that need to be considered such as the quality of schools for children, the size of the community, and the type of activities that are available.

Perhaps the most important finding in this study is that a large majority of respondents did not feel that they had enough guidance regarding the business side of the practice of orthopedics. It is clear that the training programs currently in place do not adequately prepare the residents or fellows, as half of the respondents did not remain at their first job, only 56% consulted a lawyer to review their contract, and 26% did not consult anyone.

There seems to be a large disparity in orthopedic residency and fellowship training to prepare future surgeons for the realities of practice. These are concerns that can likely be readily addressed. During the final years of orthopedic training, job evaluation and financial didactics may be provided by those in the field with experience. Future planning and consideration regarding career paths may be encouraged earlier in orthopedic training. The current study indicates that more open business discussions in training, additional training within residency or fellowship programs, and a better understanding of the healthcare system are all possible avenues of improvement. Medical student and resident career paths are influenced by their personal interests, salary potential, debt, working hours, and call responsibilities [5-7]. However, their career paths are most influenced by mentors and prior clinical experiences [8], further emphasizing the need for additional training and mentorship during residency focused on job selection.

The primary limitations of this study are inherent to its design as a survey-based study. The response rate of 19% is comparable to other physician surveys in general [9-10]. Although not all aspects of the factors influencing job selection can be studied, the current study attempted to analyze the most common. Specifically, this study did not examine burnout and its effect on the job attrition phenomenon. Additionally, the effect of global changes on the field of orthopedics and the medical landscape over time were not analyzed.

## Conclusions

When orthopedic surgeons select their first jobs, practice location, practice type, and family proximity are most important. Half of the respondents left their first post-training job, with the majority leaving within the first five years. The vast majority (86%) of survey respondents reported minimal to no education regarding job selection. It is unclear why orthopedic trainees are being poorly educated regarding job selection, and further study into the high rate of initial post-training job attrition may decrease the personal and societal costs of this phenomenon.
